# Large needle aspiration biopsy and galectin-3 determination in selected thyroid nodules with indeterminate FNA-cytology

**DOI:** 10.1038/sj.bjc.6603232

**Published:** 2006-06-27

**Authors:** A Carpi, A G Naccarato, G Iervasi, A Nicolini, G Bevilacqua, P Viacava, P Collecchi, L Lavra, C Marchetti, S Sciacchitano, A Bartolazzi

**Affiliations:** 1 Departments of Reproduction and Ageing, University of Pisa, Pisa 56024, Italy; 2 Department of Oncology, Divisions of Surgical, Molecular and Ultrastructural Pathology, University of Pisa, Pisa 56024, Italy; 3 Institute of Clinical Physiology, CNR, Pisa Italy; 4 Department of Internal Medicine, University of Pisa, Pisa 56024, Italy; 5 Department of Experimental Medicine and Endocrinology, University La Sapienza, and Ospedale San Pietro Fatebenefratelli, Associazione Fatebenefratelli per la Ricerca, Rome 00158 Italy; 6 Department of Pathology, St Andrea University Hospital, Via di Grottarossa 1035, Rome 00158, Italy; 7 Cellular and Molecular Tumor Pathology Laboratory, Cancer Center Karolinska, CCK R8:04, Karolinska Hospital, Stockholm S-17176, Sweden

**Keywords:** thyroid cancer, galectin-3, preoperative diagnosis, immunohistochemistry, cell blocks technique

## Abstract

Thyroid fine-needle aspiration biopsy (FNA)-cytology is widely used for the preoperative characterisation of thyroid nodules but this task is difficult for follicular lesions, which often remain undefined. We propose a strategy for improving the preoperative characterisation of selected follicular thyroid proliferations, which is based on large needle aspiration biopsy (LNAB) and galectin-3 expression analysis. Eighty-five thyroid specimens were obtained by LNAB (20-gauge needles) from thyroid nodules with indeterminate follicular FNA-cytology. Aspirated material was processed as a tissue microbiopsy to obtain cell blocks for both cyto/histo-morphological evaluation and galectin-3 expression analysis, by using a purified monoclonal antibody to galectin-3 and a biotin-free immunoperoxidase staining method. Preoperative diagnosis was compared to the final histology. LNAB and cell-block technique allow a preliminary distinction between nodules with a homogeneous microfollicular/trabecular structure, as frequently observed in tumours, and lesions with mixed normo–micro–macrofollicular architecture, as observed in goitre. Furthermore, LNAB provides optimal substrates for galectin-3 expression analysis. Among 85 cases tested, 14 galectin-3-positive cases were discovered preoperatively (11 thyroid cancers and three adenomas confirmed at the final histology), whereas galectin-3-negative cases were 71 (one carcinoma and 70 benign proliferations at the final histology). Sensitivity, specificity and diagnostic accuracy of this integrated morphologic and phenotypic diagnostic approach were 91.6, 97.2 and 95.3%, respectively. In conclusion, LNAB plus galectin-3 expression analysis when applied preoperatively to selected thyroid nodules candidate to surgery can potentially reduce unnecessary thyroid resections.

Preoperative characterisation of thyroid nodules is a common clinical problem. It has been estimated that about 4% of the American population between the ages of 30 and 60 years has one or more palpable thyroid nodules.

As the majority of these lesions are benign, their preoperative evaluation should be as selective as possible in the recommendation for surgical removal.

Thyroid fine-needle aspiration biopsy (FNA) is a well-established diagnostic technique, which, in the right hands, significantly reduces the number of thyroid surgical resections (Kini, 1987; Hamburger *et al*, 1979, 1989; Gharib, 1994; Carpi *et al*, 1996; Baloch *et al*, 1998; Khurana *et al*, 1999). Although FNA cytology of microfollicular nodules shows in some series a relatively high predictive value for the diagnosis of follicular neoplasms (Basu and Jayaram, 1992; Gutman and Henry, 1998), this method has important intrinsic limitations in distinguishing benign from malignant follicular lesions. This task, in fact, requires the unequivocal demonstration of capsular and/or vascular invasion, which is possible only at postoperative histology (Rosai *et al*, 1992; Bartolazzi, 2000). However, practice guidelines for the clinical management of thyroid nodules suggest that an initial FNA is more useful and cost-effective than other forms of diagnostic procedures (Hamburger *et al*, 1989). Large studies from single specialised Institutions reported a sensitivity and specificity higher than 90% leading many clinicians to recommend thyroid FNA-cytology as the initial diagnostic test in the evaluation of thyroid nodules. Surprisingly, a large prospective cohort study indicates that thyroid FNA-cytology was used as the initial diagnostic procedure in about 53% of the cases (Hundahl *et al*, 2000). Furthermore, an European survey on the clinical management of nodular thyroid lesions showed that thyroid FNA-cytology was indicated as the first diagnostic procedure by 41% of the responders, if they had to choose only one diagnostic test, and by only 25% when more than one test could be considered (Baldet *et al*, 1989). These data clearly confirm that the clinician's confidence in thyroid FNA-cytology is still low. The fact mostly reflects the large variability of thyroid FNA performance in not specialised Thyroid Hospitals and Cancer Centres.

In Community Hospitals, in fact, thyroid FNA-cytology is likely more affected by subjective evaluations, technical skills and bias of interpretations. As a consequence, the prevalence of indeterminate cytological reports indicating ‘follicular proliferations not otherwise specified’ is quite high.

In this report, we investigate whether the use of large needle aspiration biopsy (LNAB), mostly performed with 20-gauge needles, from which tissue microbiopsies and cell blocks can be prepared (Carpi *et al*, 1996, 1998, 2000), may improve the preoperative evaluation of selected follicular thyroid nodules. In addition, we wished to evaluate whether LNAB could provide better substrates for galectin-3 expression analysis compared to conventional FNA-cytology (Gasbarri *et al*, 2004).

The expression analysis of the *β*-galactosyl binding protein galectin-3 has been recently proposed for clinical use with the aim to improve the diagnostic performance of conventional FNA-cytology in distinguishing, preoperatively, benign from malignant thyroid lesions (Orlandi *et al*, 1998; Gasbarri *et al*, 1999; Inohara *et al*, 1999; Bartolazzi, 2000; Bartolazzi *et al*, 2001). Galectin-3 is undetectable in the cytoplasm of normal thyroid follicular cells, but it is almost invariably expressed in malignant transformed thyrocytes and in macrophages (Inohara *et al*, 1999; Bartolazzi *et al*, 2001). The biological role of galectin-3 expression in transformed thyrocytes is not completely understood. Interestingly, nuclear expression of galectin-3 regulates gene transcription and induces apoptosis. On the other hand, when galectin-3 accumulates in the cytoplasm of thyroid cells it blocks the apoptotic programme and let the development of cancer (Yoshii *et al*, 2001; Liu and Rabinovich, 2005). The diagnostic potential of galectin-3 expression analysis in distinguishing benign from malignant thyroid nodules has been recently evaluated in a large International Multicentric Study (Bartolazzi *et al*, 2001). Galectin-3 test method seems to be a promising diagnostic tool for improving the clinical management of patients bearing follicular thyroid proliferations. However, its clinical application is still pending (American Thyroid Association Consensus Guidelines for Thyroid Testing, 2003; Cooper *et al*, 2006).

In this study, we focus on selected follicular thyroid nodules that remained indeterminate at conventional FNA cytology. For these lesions, it was not possible to analyse galectin-3 expression on cytological preparations for scanty material and/or other technical reasons.

The diagnostic performance of LNAB plus galectin-3 expression analysis was tested on 85 follicular thyroid nodules candidate to surgery and compared with postoperative histology.

## MATERIALS AND METHODS

### Patients

Two groups of patients with follicular thyroid nodules were considered in this study.

All of them were referred to surgery because follicular lesions remained undefined after conventional FNA-cytology. A retrospective group included 65 patients, 50 women and 15 men, mean age 44 years. Forty-eight patients out of 65 were referred for a single thyroid nodule, whereas 17 out of 65 showed multiple thyroid nodules. The mean size of the single or dominant nodule object of this study was 2.66 cm (lesions ranging from 1 to 6 cm). Conventional FNA-cytology produced indeterminate reports, namely ‘follicular proliferation not otherwise specified’ in 42 of the instances, whereas presence of ‘follicular proliferations with atypical cells’ was observed in 23 of the cases. The evaluation of galectin-3 expression on FNA-material was not possible for technical reasons (poor cellularity, cell blocks not available, etc.). Large needle aspiration biopsy (mostly by using 20-gauge needles) was performed preoperatively in all the instances and galectin-3 expression analysis was applied retrospectively on LNAB-derived cell blocks.

In the prospective study, 20 patients bearing follicular thyroid nodules were subjected to preoperative LNAB plus galectin-3 expression analysis. Sixteen out of 20 patients were women, mean age 51 years. In 16 of the instances patients were referred for a single thyroid nodule whereas multiple nodules were diagnosed in four of the cases. The mean size of the single or dominant thyroid nodule object of the study was 2.36 cm (ranging from 1 to 4.3 cm). Conventional FNA-cytology resulted indeterminate in 17 out of 20 cases. Coexistence of atypical or suspicious thyroid cells was observed in two of the instances, whereas oncocytic features were detected in one case. All the patients underwent partial or total thyroidectomy and the final histological diagnosis was performed by two independent pathologists and considered as the gold standard.

### LNAB and galectin-3 test method (*galectin-3 thyrotes*t)

#### LNAB procedures

We adopted a simple and safe method of LNAB, which employs usually 20-gauge needles and exceptionally, in the largest nodules, 18-gauge needles introduced percutaneously (Carpi *et al*, 1996, 2000). When inserted into the lesion, the needle is rotated within the nodule so that its sharp end severs the tissue fragments.

The technique that was first described in 1930 (Martin and Ellis, 1930) does not show consistent differences with respect to conventional FNA-cytology (Hamburger *et al*, 1979; Carpi *et al*, 1996, 2000). Patients usually do not experience more discomfort, pain or complications than with conventional FNA method (21–25-gauge needles). Aspirated material and tissue fragments were processed to obtain paraffin-embedded cell blocks.

Formalin-fixed and paraffin-embedded cell blocks were processed to obtain seriate tissue sections for both morphologic analysis and phenotypic studies.

The morphologic evaluation of LNAB specimens let a preliminary distinction between nodules with a homogeneous microfollicular–trabecular structure, which is suggestive of tumour, from lesions with mixed normal–micro–macrofollicular architecture, which more likely represents a hyperplasic condition (goitre). Furthermore, the nuclear clearing, which represents an important feature of papillary thyroid carcinoma, can be easily appreciated in thyroid preparations from formalin-fixed and paraffin-embedded cell blocks. This is particularly important for the follicular variant of papillary carcinoma, which sometimes remains indeterminate at conventional FNA-cytology.

#### Galectin-3 test-method

A purified rat monoclonal antibody (mAb) to galectin-3 (Mabtech, Nacka, Sweden) was used in immunohistochemistry according to the manufacturer's instructions. Briefly, antigen-retrieval microwave treatment of tissues slides in 0.01 mol l^−1^ citrate buffer pH 6.0 was applied as required. To minimise the occurrence of false-positive results, a biotin-free immunoperoxidase staining method was considered in this study. This was obtained by using a horseradish-peroxidase-conjugated (HRP-conjugated) rabbit anti-rat immunoglobulins as secondary antibodies, in indirect immunoperoxidase assay (Dako, Glostrup, Denmark). Purified rat mAb directed to galectin-3 was used at a concentration range of 5–10 *μ*g/ml. After incubation with HRP-conjugated secondary antiserum, the enzymatic activity was visualised with 3,3′-diamino-benzidine (Dako).

Positive cases were classified as +/− when a heterogeneous immunostaining was restricted to isolated follicular cells or small clusters of cells, and + when the majority of thyroid cells in the follicular lesion showed galectin-3 expression. At least two experienced pathologists performed the immunohistochemical evaluation independently.

### Statistical analysis

Sensitivity, specificity, positive (PPV) and negative (NPV) predictive values, and diagnostic accuracy of the proposed immunodiagnostic method were assessed as follows:

The final histology (after surgery) was considered as the gold standard.

Sensitivity was defined on the basis of thyroid cancer immunodetection as: no. of carcinomas tested galectin-3 positive/total number of thyroid cancers. Specificity was defined on the basis of benign thyroid lesion immunodetection as: no. of benign lesions tested galectin-3 negative/total number of benign lesions. Frequency was assessed as: no. of thyroid cancers/total no. of individuals in our study. Finally, the PPV and NPV were, respectively, computed as: no. of carcinomas tested positive/total no. of tested positive cases, and no. of benign lesions tested negative/total no. of tested negative cases, respectively. Diagnostic accuracy was calculated as (no. positive+no. negative)/(true positive+false positive+true negative+false negative). STAT 6 statistical software was used to estimate the above reported parameters. This study has been carried out according to the ethical guidelines of the Declaration of Helsinki. Specific authorisation was obtained from each enrolled patient.

## RESULTS

Table 1 shows the comparative evaluation of LNAB plus galectin-3 expression analysis and the final histology in 85 follicular nodules with indeterminate FNA-cytology.

**Figure 1:**
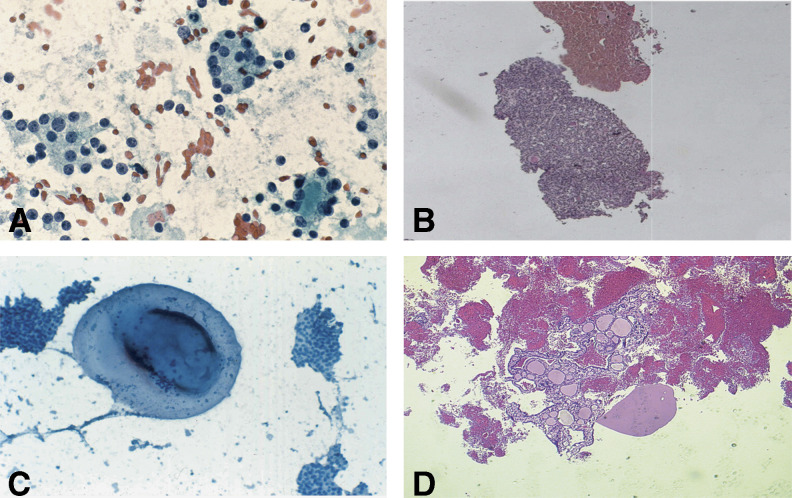
Comparative morphologic evaluation (FNA *vs* LNAB) of micro follicular/solid thyroid nodule (panels **A** and **B**) and follicular nodule with mixed architecture (panels **C** and **D**). Cell blocks/microhistology clearly provide better morphologic details of the lesions (panels **B** and **D**).

Although LNAB procedures, as well as conventional FNA cytology, fail to distinguish between benign and malignant follicular lesions, the former provides a better sampling of the lesion, with more aspirated material and improved architectural view (Figure 1).

In fact, a careful morphologic evaluation of the collected LNAB material let us a preliminary distinction of the follicular thyroid nodules in two major groups: (a) follicular proliferations with homogeneous microfollicular/solid or trabecular architecture, which suggests a thyroid neoplasia, and (b) follicular lesions with mixed micro–normal–macrofollicular structure, which more likely suggests a nodular hyperplasia (thyroid goitre).

As reported in Table 1, nodules showing at LNAB a homogeneous microfollicular/solid architecture were observed in 39 out of 85 instances (45.9%). In about 50% of these cases, the final histology confirmed the presence of benign or malignant tumours: 13 adenomas (33.3% of the cases) and six carcinomas (15.4%). Nodules with mixed follicular structure were detected in 45 out of 85 cases tested (52.9%). Postoperative histology showed thyroid hyperplasia in 34 of the instances (75.5% of the cases). In this group of lesions, six (13.3%) adenomas were histologically detected whereas thyroid malignancies, which were invariably represented by follicular variant of papillary carcinomas, were five (11.1%). The proportion of suspected neoplastic or hyperplasic nodules between the two groups of follicular proliferations, as evaluated by preoperative LNAB, was statistically significant (*χ*^2^=4.36; *P*=0.0244).

**Figure 2:**
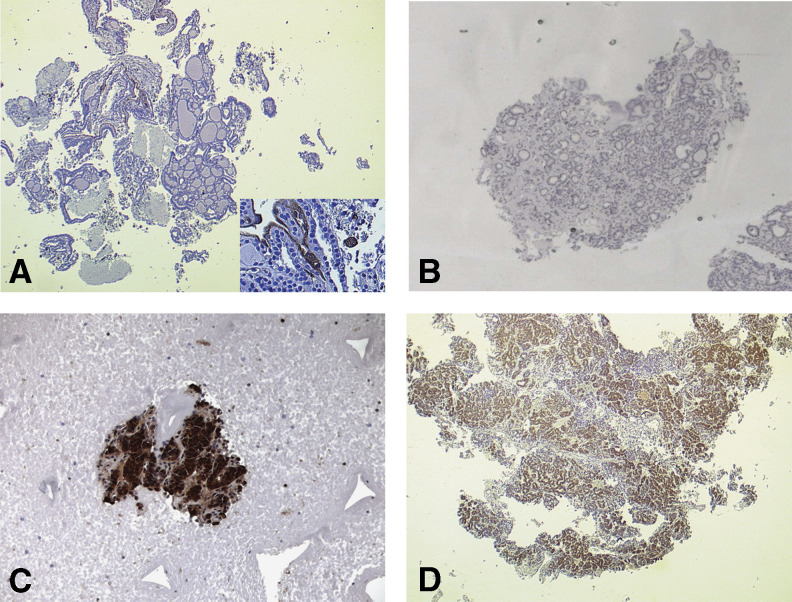
Galectin-3 thyrotest negative on follicular hyperplasia (panel **A**). The inset shows endothelial cells and one foamy macrophage galectin-3 positive as internal controls. A microfollicular adenoma galectin-3 negative (panel **B**). Solid/trabecular (panel **C**) and micro follicular (panel **D**) thyroid malignancies, both of which are immunoreactive for galectin-3 (indirect immunoperoxidase biotin-free).

The immunophenotypic analysis of microfollicular/solid nodules evaluated on LNAB specimens, showed galectin-3 expression in five out of 39 cases (Figure 2). All of them were finally diagnosed as thyroid malignancies (Table 1). Postoperative histology demonstrated, in fact, the presence of two follicular variants of papillary carcinoma, a papillary carcinoma with solid/poorly differentiated areas, a follicular carcinoma with oncocytic features and one conventional follicular carcinoma. The latter showed a heterogeneous expression of galectin-3, with several negative neoplastic cells.

Among the 34 galectin-3-negative microfollicular/solid lesions, 13 were histologically classified as follicular adenomas (39.3%), 20 as nodular hyperplasias (58.8%) and one as follicular carcinoma. The latter should be considered a true galectin-3-negative carcinoma because the lack of expression of galectin-3 was confirmed by immunohistochemistry after surgery.

On the other hand, among the 45 LNAB cases with mixed follicular architecture, which were suspicious for benign thyroid conditions, postoperative histology confirmed hyperplasia in 34 of the cases (75.5%). Interestingly, all of them were invariably galectin-3 negative. It is noteworthy that all of the five thyroid carcinomas in this group were immunoreactive with the mAb to galectin-3. Galectin-3 expression was also detected in three out of six histologically confirmed follicular adenomas. Two of these lesions were finally diagnosed as ‘follicular proliferations with undefined malignant potential (UMP) (Rosai *et al*, 1992; Papotti *et al*, 2005) because evidence of capsular impingement of the neoplastic cells, without clear aspects of invasion, was histologically demonstrated after evaluation of multiple tissue sections.

In one instance, LNAB material showed galectin-3-positive atypical cells. Also in this case the final histological report confirmed the presence of a papillary carcinoma.

Sensitivity and specificity of this integrated diagnostic approach were 91.6 and 97.2%, respectively. The PPV was 78.4% (>92% if galectin-3-positive follicular proliferations UMP are considered as early transformed lesions or ‘thyroid cancer precursor lesions’) (Bartolazzi *et al*, 2001; Papotti *et al*, 2005), whereas the NPV was 98.6%. Diagnostic accuracy assessed in this study was 95.3%, and frequency 14.1.

## DISCUSSION

FNA-cytology is widely used for the preoperative evaluation of thyroid nodules, but its optimal diagnostic performance is restricted to thyroid specialised hospitals and cancer centres. However, it is well known that thyroid FNA-cytology has *per se* important intrinsic limitations in distinguishing benign from malignant follicular lesions (Kini, 1987; Rosai *et al*, 1992; Gharib, 1994). Even in *‘the right hands’* the rate of inadequate smears is rarely lower than 10%, and up to 30% of FNA cytological reports remain undefined (Kini, 1987; Rosai *et al*, 1992; Gharib, 1994). For this reason, the characterisation of follicular thyroid nodules is widely considered as the ‘grey zone of FNA-cytology’. Many authors reported that high cellularity, nuclear features, scanty colloid and presence of microfollicular structures are the principal morphologic features to be considered for a better cytological diagnosis of a follicular neoplasm (Rojeski and Gharib, 1985; Kini, 1987; Hamburger *et al*, 1989; Gharib, 1994; Baloch *et al*, 1998; Gutman and Henry, 1998). Combination of high cellularity, microfollicular/solid pattern and nuclear atypia are predictive of a follicular neoplasm in the majority of the instances (Basu and Jayaram, 1992; Solomon, 1993; Carpi *et al*, 2000) but these features are not sufficient for distinguishing between benign and malignant lesions (Mazzaferri, 1993; Greaves *et al*, 2000).

We and other laboratories have recently proposed the use of galectin-3 expression analysis for the preoperative characterisation of follicular thyroid nodules (Orlandi *et al*, 1998; Gasbarri *et al*, 1999; Inohara *et al*, 1999; Bartolazzi *et al*, 2001; Herrmann *et al*, 2002; Collet *et al*, 2005). Although many reports have been published on this issue during the last decade (about 100 papers are displayed in Pub Med since 1997) some authors experienced conflicting results with the use of galectin-3 for improving diagnosis of follicular thyroid proliferations (Martins *et al*, 2002; Niedziela *et al*, 2002; Feilchenfeldt *et al*, 2003; Jakubiak-Wielganowicz *et al*, 2003; De-Leon-Mazariegos *et al*, 2004; Mehrotra *et al*, 2004; Mills *et al*, 2005). However, some of these reports show major methodological problems (Herrmann *et al*, 2002; Bartolazzi *et al*, 2003; Bartolazzi and Bussolati, 2006). The use of reverse transcriptase (RT-PCR)–polymerase chain reaction technique for evaluation of galectin-3 expression in thyroid nodules, in fact, can produce false positive results because foamy thyroid macrophages, endothelial cells and some activated lymphocytes normally express galectin-3 (Bartolazzi *et al*, 2003). Furthermore, the use of purified monoclonal antibodies to galectin-3 in a biotin-free detection system is advisable in order to reduce the occurrence of false-positive results. This may be important when galectin-3 test method is used for the diagnostic purpose (Herrmann *et al*, 2002; Bartolazzi *et al*, 2003; Bartolazzi and Bussolati, 2006).

Although galectin-3 test method may be further optimised in order to be widely used in the clinical practice on conventional cytological preparations, the results of a large prospective multicentric study, which is running at national level, seems to confirm its diagnostic value (work in preparation).

HBME1, CK19, CD26/DPPIV, TPO and c-met have also been reported to be useful markers for improving the preoperative characterisation of thyroid nodules (De Micco *et al*, 1994, 1999; Ruco *et al*, 2001; Aratake *et al*, 2002; Rosai, 2003; Maruta *et al*, 2004; Mechanick, 2004; Weber *et al*, 2004; Papotti *et al*, 2005; Prasad *et al*, 2005; Saggiorato *et al*, 2005; de Matos *et al*, 2005). Among the aforementioned molecules, TPO is the largest preoperatively investigated tumour marker with a high overall accuracy (De Micco *et al*, 1994, 1999; Christensen *et al*, 2000; Weber *et al*, 2004). However, for the majority of these molecules the biological rationale of their preferential expression in thyroid cancer is still missing.

Anyway it should be stressed that LNAB and cell-block technique can eventually provide optimal substrates for comparative immunohisto-cytochemistry (on seriate tissue sections) directed to evaluate the expression of galectin-3 and other potential markers of thyroid cancer. This analysis is almost impossible to be performed on conventional cytological smears.

In this report, we show that LNAB plus galectin-3 determination led to correctly identify almost all of the benign thyroid nodules (97%), which were considered as suspicious following conventional FNA cytology. If it is true that FNA-derived cellular specimens are obtained and handled more easily and cheaply than LNAB-derived small tissue fragments, it should be mentioned that the occurrence of inadequate cytological specimens is higher with FNA than with LNAB preparations obtained with 20-gauge needle (Carpi *et al*, 1996, 1998). It has been recently reported that about 23% of thyroid aspirates have a low cellularity and are not suitable for cell-block preparation and galectin-3 immunostaining (Mills *et al*, 2005). In our experience, LNAB performed with 20-gauge needle retrieves much more tissue than FNA to be processed as paraffin-embedded cell block (Gasbarri *et al*, 2004). Most important, LNAB-derived cell blocks remain available for repetition of the galectin-3 test as well as for testing of different markers at any time.

Thyroid LNAB is safe as reported in series of thousands consecutive examinations (Crile and Hawk, 1973; Hamburger *et al*, 1979; Carpi *et al*, 1996, 2000). One of us (A Carpi) performed more than 3000 LNABs in the last decade with a few (about 10) minor complications consisting in relatively large haematomas with spontaneous resolution. However, the purpose of this method is not for routine cyto/histological evaluation of thyroid nodules, but it should be restricted to those selected lesions referred to surgery because indeterminate at conventional FNA-cytology. In our series, a preliminary distinction between neoplastic nodules (benign or malignant) and hyperplasia (goitre) was possible on morphological bases alone in more than 73% of the cases, by using cell-block preparations conventionally stained with haematoxylin and eosin (microhistology). When *galectin-3 thyrotest* was applied on the same substrates, the diagnostic accuracy of the method improved consistently up to 95%. Interestingly, among the 12 thyroid carcinomas detected at the final histology, only one did not express galectin-3. However, this lesion, although indeterminate at conventional FNA-cytology, was considered morphologically suspicious for neoplasia, because a microfollicular structure with clear nuclei was evident at LNAB microhistology (paraffin-embedded and formalin-fixed tissue section). Most important, none of the histologically confirmed hyperplasic conditions (goitres) showed expression of galectin-3. Therefore, the NPV of the proposed integrated diagnostic method is very high (>97%) when the galectin-3 test method is correctly applied (Bartolazzi *et al*, 2001, 2003; Bartolazzi and Bussolati, 2006).

Regarding the detection of some galectin-3-positive follicular adenomas, we previously reported that these lesions may likely represent early follicular carcinomas, in which capsular and/or vascular invasion cannot be demonstrated yet (Bartolazzi *et al*, 2001).

In this study, we detected three galectin-3-positive follicular adenomas out of 19 tested.

We believe that surgical excision of these specific lesions may be advisable and does not represent a real overtreatment. In fact, the expression of galectin-3 in thyroid cells drives the block of apoptosis, a biological feature that is not observed in normal cells (Yoshii *et al*, 2001; Takenaka *et al*, 2003; Liu and Rabinovich, 2005).

In our series, the number of follicular cancers is limited for evaluating galectin-3 diagnostic sensitivity for these specific malignancies. However, data from us and other laboratories show that galectin-3 test method works well also in follicular carcinomas (Bartolazzi *et al*, 2001; Oestreicher-Kedem *et al*, 2004; Collet *et al*, 2005; Papotti *et al*, 2005; Saggiorato *et al*, 2005). By the practical point of view, the incidence of well-differentiated follicular carcinomas is low (less than 10% of the cases) and is markedly reduced in countries without iodine deficiency. In fact, in many series including excised nodules with indeterminate FNA cytology, the follicular variant of papillary carcinoma is the most common thyroid malignancy detected at histology (Hooft *et al*, 2004; Miller *et al*, 2004; Carpi *et al*, 2005).

In conclusion, thyroid LNAB when integrated with galectin-3 expression analysis provides a potent and reliable diagnostic tool for improving the clinical management of patients bearing follicular thyroid nodules. The purpose of LNAB and galectin-3 expression analysis is not to replace conventional thyroid FNA-cytology, which remains in our opinion, the most important screening method for thyroid proliferations. The combined diagnostic approach we propose should be applied in all the instances in which FNA is inadequate for technical or logistic reasons and *galectin-3 thyrotest* cannot be performed on FNA-derived material. LNAB-derived cell blocks and galectin-3 test method let a better preoperative selection of patients candidate to surgery. A consistent reduction of hospitalisation and social costs are expected when most of the unnecessary surgery for benign thyroid nodules will be avoided.

## Figures and Tables

**Table 1:** Preoperative LNAB plus Galectin-3 expression analysis *vs* postoperative histology

**Preoperative diagnosis**			**Postoperative histology**		
**LNAB**	**Galectin-3 expression**		**Cancer**	**Adenoma**	**Hyperplasia**
Microfollicular/solid 39 (45.9%)	Gal-3+	5	5	0	0
	Gal-3−	34	1	13 (33.3%)	20 (59.3%)
Atypical cells 1	Gal-3+	1	1	0	0
	Gal-3−	0	0	0	0
Follicular/mixed architecture 45 (52.9%)	Gal-3+	8	5	3^a^	0
	Gal-3−	37	0	3	34 (75.5%)
					
Total 85		*85*	12	19	54

a Two of these galectin-3-positive adenomas showed capsular impingement of neoplastic cells. %=percentage in Table 1 are referred to the total number of cases.

## References

[bib1] Aratake Y, Umeki K, Kiyoyama K, Hinoura Y, Sato S, Ohno a, Kuribayashi T, Hirai K, Nabeshima K, Kotani T (2002) Diagnostic utility of galectin-3 and CD26/DPPIV as preoperative diagnostic markers for thyroid nodules. Diagn Cytopathol 26: 366–372 12112826 10.1002/dc.10111

[bib2] Baldet L, Manderscheid JC, Glinoer D, Jaffiol C, Coste-Seignovert B, Percheron C (1989) The management of differentiated thyroid cancer in Europe in 1988. Results of an international survey. Acta Endocrinol 120: 547–558 10.1530/acta.0.1200547 2728801

[bib3] Baloch ZW, Sack MJ, Yu GH, Livolsi VA, Gupka PK (1998) Fine-needle aspiration of thyroid: an institutional experience. Thyroid 8: 565–569 9709908 10.1089/thy.1998.8.565

[bib4] Basu D, Jayaram G (1992) A logistic model for thyroid lesions. Diagn Cytopathol 8: 23–27 1551363 10.1002/dc.2840080106

[bib5] Bartolazzi A (2000) Improving accuracy of cytology for nodular thyroid lesions. The Lancet 355: 1661–1662 10.1016/S0140-6736(00)02233-9 10905237

[bib6] Bartolazzi A, Bussolati G (2006) Galectin-3 does not reliably distinguish benign from malignant thyroid neoplasms. Histopathology 48: 212–213 16405677 10.1111/j.1365-2559.2005.02214.x

[bib7] Bartolazzi A, Gasbarri A, Papotti M, Bussolati G, Lucante T, Kahan A, Inohara H, Marandino F, Orlandi F, Nardi F, Vecchione A, Tecce R, Larsson O, the Thyroid Cancer Study Group (2001) Application of an immunodiagnostic method for improving preoperative diagnosis of nodular thyroid lesions. Lancet 357: 1644–1650 11425367 10.1016/s0140-6736(00)04817-0

[bib8] Bartolazzi A, Papotti M, Orlandi F (2003) Methodological considerations regarding the use of galectin-3 expression analysis in preoperative evaluation of thyroid nodules. J Clin Endocrinol Metab 88: 950–951 12574240 10.1210/jc.2002-021593

[bib9] Carpi A, Ferrari E, Toni MG, Sagripanti A, Nicolini A, Di Coscio G (1996) Needle aspiration techniques in preoperative selection of patients with thyroid nodules: a long-term study. J Clin Oncol 14: 1704–1712 8622091 10.1200/JCO.1996.14.5.1704

[bib10] Carpi A, Nicolini A, Gross MD, Fig LM, Shapiro B, Fanti S, Rampin L, Polico C, Rubello D (2005) Controversies in diagnostic approaches to the indeterminate follicular thyroid nodule. Biomed Parmacother 59: 517–520 10.1016/j.biopha.2005.04.003 16202555

[bib11] Carpi A, Nicolini A, Sagripanti A, Righi C, Fabris FM, Di Coscio G (2000) Large-needle aspiration biopsy for the preoperative selection of palpable thyroid nodules diagnosed by fine-needle aspiration as micro follicular nodule or suspected cancer. Am J Clin Pathol 113: 872–877 10874889 10.1309/JCU6-Y4DC-LEVM-HBFJ

[bib12] Carpi A, Sagripanti A, Nicolini A, Santini S, Ferrari E, Romani R, Di Coscio G (1998) Large needle aspiration biopsy for reducing the rate of inadequate cytology on fine needle aspiration specimens from palpable thyroid nodules. Biomed Pharmacother 52: 303–307 9809173 10.1016/s0753-3322(98)80025-5

[bib13] Christensen L, Blichert-Toft M, Brandt M, Lange M, Sneppen SB, Ravnsbaek J, Mollerup CL, Strange L, Jensen F, Kirkegaard J, Sand Hansen H, Sorensen SS, Feldt-Rasmussen U (2000) Thyroperoxidase (TPO) immunostaining of the solitary cold thyroid nodule. Clin Endocrinol 53: 161–169 10.1046/j.1365-2265.2000.01035.x 10931096

[bib14] Collet JF, Hurbain I, Prengel C, Utzmann O, Scetbon F, Bernaudin JF, Fajac A (2005) Galectin-3 immunodetection in follicular thyroid neoplasms: a prospective study on fine-needle aspiration samples. Br J Cancer 93: 1175–1181 16251880 10.1038/sj.bjc.6602822 PMC2361502

[bib15] Cooper DS, Doherty GM, Haugen BR, Kloos RT, Lee SL, Mandel SJ, Mazzaferri EL, Mclver B, Sherman SI, Lee SL, Tuttle RM (2006) Management guidelines for patients with thyroid nodules and differentiated thyroid cancer. Thyroid 16: 1–33 16420177 10.1089/thy.2006.16.109

[bib16] Crile Jr GF, Hawk Jr WA (1973) Aspiration biopsy of thyroid nodules. Surg Gynecol Obstet 136: 241 4739351

[bib17] De-Leon-Mazariegos R, Canedo-Patzi M, Perez-Enriquez B, Candanedo-Gonzales F, Saqui-Salces M, Gamboa-Dominguez A, Rull-Rodrigo JA (2004) Low galectin-3 capacity to discriminate thyroid lesions. Rev Invest Clin 56: 623–628 15776867

[bib18] De Matos PS, Ferreira AP, de Oliveira Facuri F, Assumpcao LV, Metze K, Ward LS (2005) Usefulness of HBME-1 cytokeratin 19 and galectin-3 immunostaining in the diagnosis of thyroid malignancy. Histopathology 47: 391–401 16178894 10.1111/j.1365-2559.2005.02221.x

[bib19] De Micco C, Vasko V, Garcia S, Zoro P, Denizot A, Henry JF (1994) Malignancy markers in the cytodiagnosis of thyroid nodules. Fine-needle aspiration of thyroid follicular neoplasm: diagnostic use of thyroid peroxidase immunocytochemistry with monoclonal antibody 47. Surgery 116: 1031–1035 7985083

[bib20] De Micco C, Vasko V, Henry JF (1999) The value of thyroid peroxidase immunohistochemistry for preoperative fine-needle aspiration diagnosis of the follicular variant of papillary thyroid cancer. Surgery 126: 1200–1204 10598208 10.1067/msy.2099.101428

[bib21] Feilchenfeldt J, Totsch M, Sheu SY, Robert J, Spiliopoulos A, Frilling A, Schmid KW, Meier CA (2003) Expression of galectin-3 in normal and malignant thyroid tissues by quantitative PCR and immunohistochemistry. Mod Pathol 16: 1117–1123 14614051 10.1097/01.MP.0000096047.99202.31

[bib22] Gasbarri A, Martegani MP, Del Prete F, Lucante T, Natali PG, Bartolazzi A (1999) Galectin-3 and CD44v6 isoforms in the preoperative evaluation of thyroid nodules. J Clin Oncol 17: 3494–3502 10550147 10.1200/JCO.1999.17.11.3494

[bib23] Gasbarri A, Marchetti C, Iervasi G, Bottoni A, Nicolini A, Bartolazzi A, Carpi A (2004) From the bench to the bedside. Galectin-3 immunodetection for improving the preoperative diagnosis of the follicular thyroid nodules. Biomed Pharmacother 58: 356–359 15271416 10.1016/j.biopha.2004.05.004

[bib24] Gharib H (1994) Fine-needle aspiration biopsy of thyroid nodules: Advantages, limitations and effects. Mayo Clin Proc 69: 44–50 8271850 10.1016/s0025-6196(12)61611-5

[bib25] Greaves TS, Olvera M, Florentine BD, Raza AS, Cobb CJ, Tsao-Wei DD, Groshen S, Singer P, Lopresti JJ, Martin SE (2000) Follicular lesions of thyroid. A 5 years fine needle aspiration experience. Cancer Cytopath 90: 335–341 11156516

[bib26] Gutman PD, Henry M (1998) Fine needle aspiration cytology of the thyroid. Clin Lab Med 18: 461–482 9742379

[bib27] Hamburger IL, Miller JM, Kini SR (1979) Pathological evaluation of thyroid nodules. In Handbook and Atlas, Southfield (eds). MI, USA: Southfield

[bib28] Hamburger JI, Husain M, Nishiyama R, Nunez C, Solomon D (1989) Increasing the accuracy of fine-needle biopsy for thyroid nodules. Arch Pathol Lab Med 113: 1035–1041 2774855

[bib29] Herrmann MF, LiVolsi VA, Pasha TL, Roberts SA, Wojcik EM, Baloch ZW (2002) Immunohistochemical expression of galectin-3 in benign and malignant thyroid lesions. Arch Pathol Lab Med 126: 710–713 12033961 10.5858/2002-126-0710-IEOGIB

[bib30] Hooft L, Hoekstra OS, Boers M, Van Tulder MW, Van Diest P, Lips P (2004) Practice, efficacy, and costs of thyroid nodule evaluation: a retrospective study in a Dutch university hospital. Thyroid 14: 287–293 15142362 10.1089/105072504323030942

[bib31] Hundahl SA, Cady B, Cunningham MP, Mazzaferri E, McKee RF, Rosai J, Shah JP, Fremgen AM, Stewart AK, Holzer S (2000) Initial results from a prospective cohort study of 5583 cases of thyroid carcinoma treated in the United States during 1996. Cancer (Cytopathol) 89: 202–217 10.1002/1097-0142(20000701)89:1<202::aid-cncr27>3.0.co;2-a 10897019

[bib32] Inohara H, Honjo Y, Yoshii T, Akahani S, Yoshida J, Hattoro K, Okamoto S, Sawada T, Raz A, Kubo T (1999) Expression of galectin-3 in fine-needle aspirates as a diagnostic marker differentiating benign from malignant thyroid neoplasms. Cancer 85: 2475–2484 10357421

[bib33] Jakubiak-Wielganowicz M, Kubiak R, Sygut J, Pomorski L, Kordek R (2003) Usefulness of galectin-3 immunohistochemistry in differential diagnosis between thyroid follicular carcinoma and follicular adenoma. Pol J Pathol 54: 11–115 14575419

[bib34] Khurana KK, Labrador E, Izquierdo R, Mesonero CE, Pisharodi LR (1999) The role of fine-needle aspiration biopsy in the management of thyroid nodules in children, adolescents and young adults: a multi-institutional study. Thyroid 4: 383–386 10.1089/thy.1999.9.383 10319945

[bib35] Kini SR (1987) Thyroid. In Guide to Clinical Aspiration Biopsy, Kline TS (ed), Vol. 3. New York: Igaku-Shoin

[bib36] Liu F, Rabinovich GA (2005) Galectins as modulators of tumor progression. Nat Rev Cancer 5: 29–41 15630413 10.1038/nrc1527

[bib37] Martin HE, Ellis EB (1930) Biopsy by needle puncture and aspiration. Ann Surg 92: 169–181 17866350 10.1097/00000658-193008000-00002 PMC1398218

[bib38] Martins L, Matsuo SE, Ebina KN, Kulcsar MA, Friguglietti CU, Kimura ET (2002) Galectin-3 messenger ribonucleic acid and protein are expressed in benign thyroid tumors. J Clin Endocrinol Metab 87: 4806–4810 12364477 10.1210/jc.2002-020094

[bib39] Maruta J, Hashimoto H, Yamashita H, Noguchi S (2004) Diagnostic applicability of dipeptidyl aminopeptidase IV activity in cytological samples for differentiating follicular thyroid carcinoma from follicular adenoma. Arch Surg 139: 83–88 14718282 10.1001/archsurg.139.1.83

[bib40] Mazzaferri EL (1993) Management of a solitary thyroid nodule. N Engl J Med 328: 553–559 8426623 10.1056/NEJM199302253280807

[bib41] Mehrotra P, Okpokam A, Bouhaidar R, Johnson SJ, Wilson JA, Davies BR, Lennard TW (2004) Galectin-3 does not reliably distinguish benign from malignant thyroid neoplasms. Histopathology 45: 493–500 15500653 10.1111/j.1365-2559.2004.01978.x

[bib42] Mechanick JI (2004) Diagnosis and management of thyroid nodules. In: Endocrine Surgery, Schwartz AE, Pertsemlidis D, Gagner M (eds) pp 115–131. New York: Marcel Dekker, Inc

[bib43] Miller B, Burkey S, Lindberg G, Snyder WH rd, Nwariaku FE (2004) Prevalence of malignancy within cytologically indeterminate thyroid nodules. Am J Surg 188: 459–462 15546550 10.1016/j.amjsurg.2004.07.006

[bib44] Mills LJ, Poller DN, Yiangou C (2005) Galectin-3 is not useful in thyroid FNA. Cytopathology 16: 132–138 15924608 10.1111/j.1365-2303.2005.00213.x

[bib45] Niedziela M, Maceluch J, Korman E (2002) Galectin-3 is not an universal marker of malignancy in thyroid nodular disease in children and adolescents. J Clin Endocrinol Metabol 87: 4411–4415 10.1210/jc.2002-020387 12213909

[bib46] Oestreicher-Kedem Y, Halpern MM, Roizman P, Hardy B, Sulkes J, Feinmesser R, Stern Y (2004) Diagnostic value of galectin-3 as a marker for malignancy in follicular patterned thyroid lesions. Head Neck 26: 960–966 15386597 10.1002/hed.20087

[bib47] Orlandi F, Saggiorato E, Pivano G, Puligheddu B, Termine A, Cappia S, De Giuli P, Angeli A (1998) Galectin-3 is a presurgical marker of human thyroid carcinoma. Cancer Res 58: 301–310 9679965

[bib48] Papotti M, Rodriguez J, De Pompa R, Bartolazzi A, Rosai J (2005) Galectin-3 and HBME-1 expression in well-differentiated thyroid tumors with follicular architecture of uncertain malignant potential. Mod Pathol 18: 541–546 15529186 10.1038/modpathol.3800321

[bib49] Prasad ML, Pellegata NS, Huang Y, Nagaraja HN, de la Chapelle A, Kloos RT (2005) Galectin-3 fibronectin-1, CITED-1, HBME1 and cytokeratin-19 immunohistochemistry is useful for differential diagnosis of thyroid tumors. Mod Pathol 18: 48–57 15272279 10.1038/modpathol.3800235

[bib50] Rosai J (2003) Immunohistochemical markers of thyroid tumors: significance and diagnostic applications. Tumori 89: 517–519 14870775 10.1177/030089160308900511

[bib51] Rosai J, Carcangiu ML, De Lellis RA (1992) Atlas of Tumor Pathology: Tumors of the Thyroid Gland, Third series, fascicles 5 Washington, DC (USA): Armed Force Institute of Pathology

[bib52] Rojeski MT, Gharib H (1985) Nodular thyroid disease: evaluation and management. N Engl J Med 313: 428–436 3894966 10.1056/NEJM198508153130707

[bib53] Ruco PL, Stoppacciaro A, Ballarini F, Prat M, Scarpino S (2001) Met protein and hepatocyte growth factor (HGF) in papillary carcinoma of the thyroid: evidence for a pathogenetic role in tumorigenesis. J Pathol 194: 4–8 11329134 10.1002/path.847

[bib54] Saggiorato E, De Pompa R, Volante M, Cappia S, Arecco F, Dei Tos AP, Orlandi F, Papotti M (2005) Characterization of thyroid ‘follicular neoplasms’ in fine-needle aspiration cytological specimens using a panel of immunohistochemical markers: a proposal for clinical application. Endocr Relat Cancer 12: 305–317 15947105 10.1677/erc.1.00944

[bib55] Solomon D (1993) Fine needle aspiration of the thyroid: an update. Thyroid today 16: 1–9

[bib56] Takenaka Y, Inohara H, Yoshii T, Oshima K, Nakahara S, Akahani S, Honjo Y, Yamamoto Y, Raz A, Kubo T (2003) Malignant transformation of thyroid follicular cells by galectin-3. Cancer Lett 30: 111–119 10.1016/s0304-3835(03)00056-9 12767519

[bib57] The American Thyroid Association, Consensus Guidelines for Thyroid Testing in the New Millennium (2003) In Laboratory Medicine Practice Guidelines. Laboratory support for the diagnosis and monitoring of thyroid disease. (Section) H: thyroid fine needle aspiration (FNA) and cytology. Thyroid (monography) 13: 80–86

[bib58] Weber KB, Shroyer KR, Heinz DE, Nawaz S, Said MS, Haugen BR (2004) The use of combination of galectin-3 and thyroid peroxidase for the diagnosis and prognosis of thyroid cancer. Am J Clin Pathol 122: 524–531 15487449 10.1309/UUQT-E505-PTN5-QJ7M

[bib59] Yoshii T, Inohara H, Takenaka Y, Honjo Y, Akahani S, Nomura T, Raz A, Kubo T (2001) Galectin-3 maintains the transformed phenotype of thyroid papillary carcinoma cells. Int J Oncol 18: 787–792 11251175 10.3892/ijo.18.4.787

